# Supplementary material for: Generative Design of Cell Type-Specific RNA Splicing Elements for Programmable Gene Regulation
Source: bioRxiv. 2025 Nov 5:2025.11.05.686847. Preprint. [Version 1] doi: 10.1101/2025.11.05.686847 (PMC12637576; doi:10.1101/2025.11.05.686847)
Supplement: 4 [file NIHPP2025.11.05.686847v1-supplement-4.pdf]

bioRxiv preprint doi: https://doi.org/10.1101/2025.11.05.686847; this version posted November 5, 2025. The copyright holder for this preprint (which was not certified by peer review) is the author/funder, who has granted bioRxiv a license to display the preprint in perpetuity. It is made available under aCC-BY 4.0 International license.

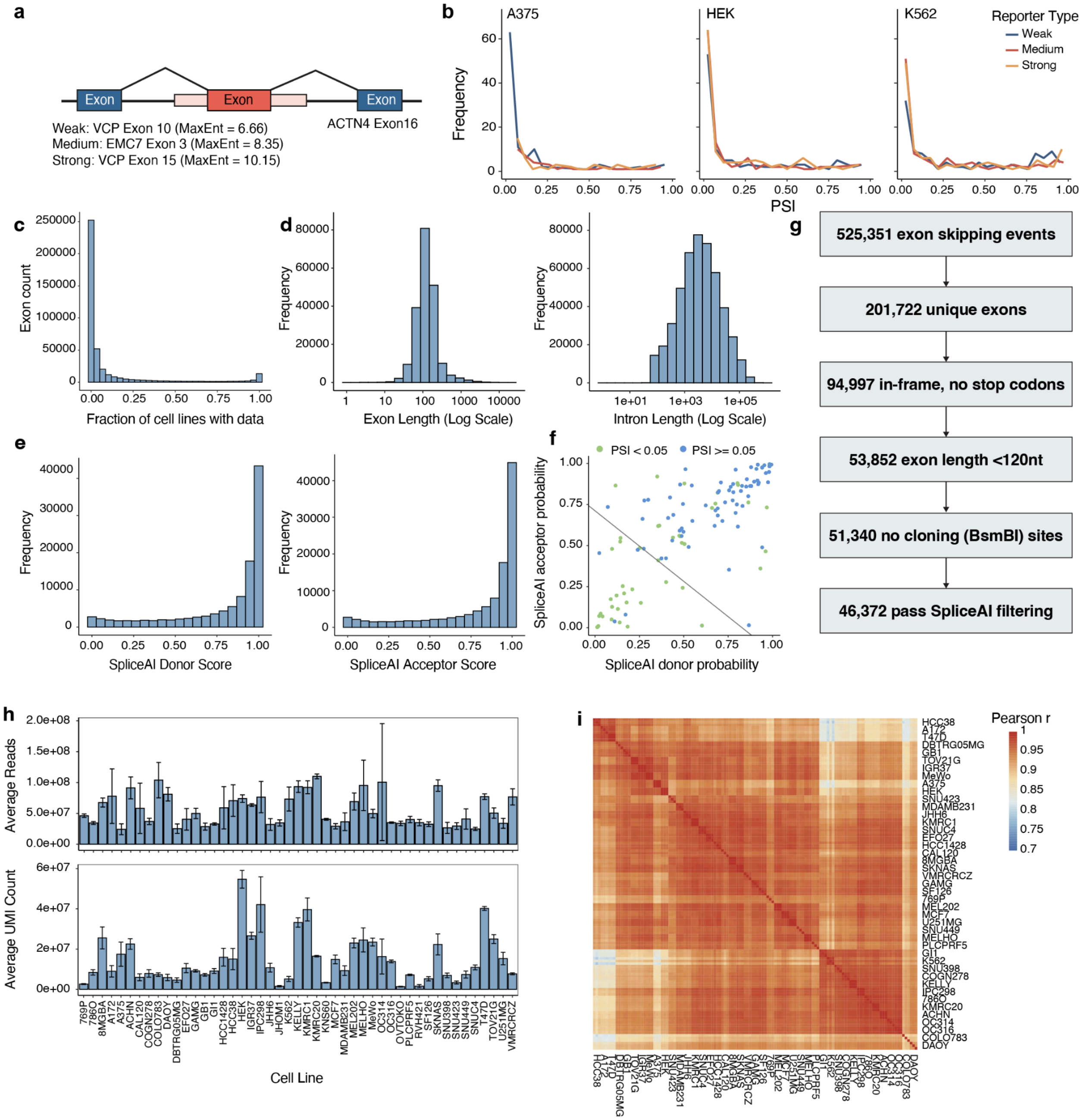

Supplementary Figure 1: Reporter design, library construction, and data quality control.

a, Exon skipping reporter constructs with weak (*VCP* Exon 10, MaxEntScan = 6.66), medium (*EMC7* Exon 3, MaxEntScan = 8.35), or strong (*VCP* Exon 15, MaxEntScan = 10.15) 5′ splice sites. All constructs contain *ACTN4* Exon 16 as the constant downstream exon.

b, PSI distributions across weak, medium, and strong reporter backbones in A375, HEK293, and K562.

c, Fraction of CCLE cell lines with quantifiable exon skipping events from RNA-seq.

bioRxiv preprint doi: https://doi.org/10.1101/2025.11.05.686847; this version posted November 5, 2025. The copyright holder for this preprint (which was not certified by peer review) is the author/funder, who has granted bioRxiv a license to display the preprint in perpetuity. It is made available under aCC-BY 4.0 International license.

d, Exon length (left) and intron length (right) distributions for candidate library elements.
e, Distribution of SpliceAI-predicted donor (left) and acceptor (right) scores for candidate exons.
f, Scatterplot of SpliceAI donor and acceptor probabilities, colored by PSI measured in pilot experiments.
g, Filtering workflow for construction of the final exon library.
h, Average reads (top) and unique molecular identifier (UMI) counts (bottom) per cell line. Error bars show standard deviation across replicates.
i, Pairwise Pearson's correlation coefficients of exon PSI values between replicates across all profiled cell lines.

bioRxiv preprint doi: https://doi.org/10.1101/2025.11.05.686847; this version posted November 5, 2025. The copyright holder for this preprint (which was not certified by peer review) is the author/funder, who has granted bioRxiv a license to display the preprint in perpetuity. It is made available under aCC-BY 4.0 International license.

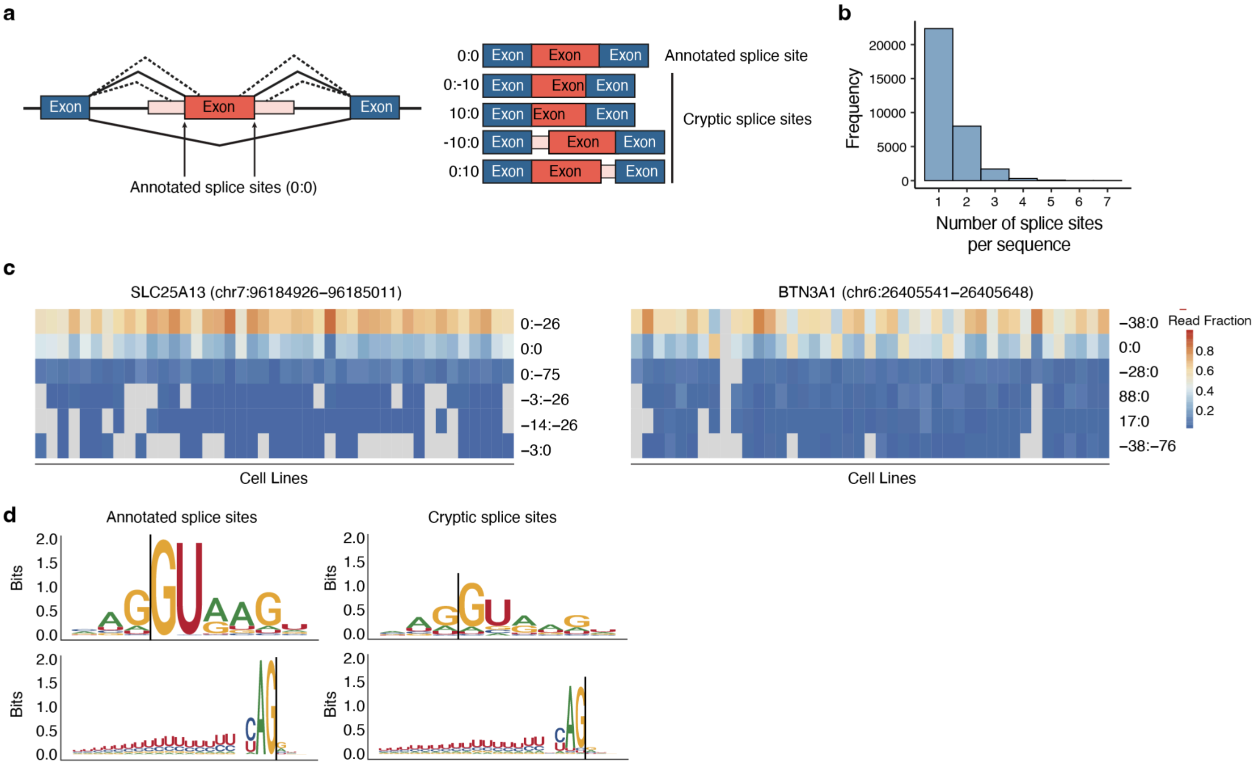

Supplementary Figure 2: Cryptic and annotated splice site usage across library elements
a, Schematic of annotated versus cryptic splice site usage within the exon skipping reporter system. Cryptic splice sites use non-annotated 3′ or 5′ sites, and can result in additional or fewer nucleotides at each end of the library exon.
b, Histogram of the number of splice sites detected per library element.
c, Representative examples of library elements with multiple splice sites used consistently across cell lines, showing annotated and cryptic splice site junction usage.
d, Sequence logos of annotated donor (5′, top left) and acceptor (3′, top right) splice sites, and cryptic donor (5′, bottom left) and acceptor (3′, bottom right) splice sites.

bioRxiv preprint doi: https://doi.org/10.1101/2025.11.05.686847; this version posted November 5, 2025. The copyright holder for this preprint (which was not certified by peer review) is the author/funder, who has granted bioRxiv a license to display the preprint in perpetuity. It is made available under aCC-BY 4.0 International license.

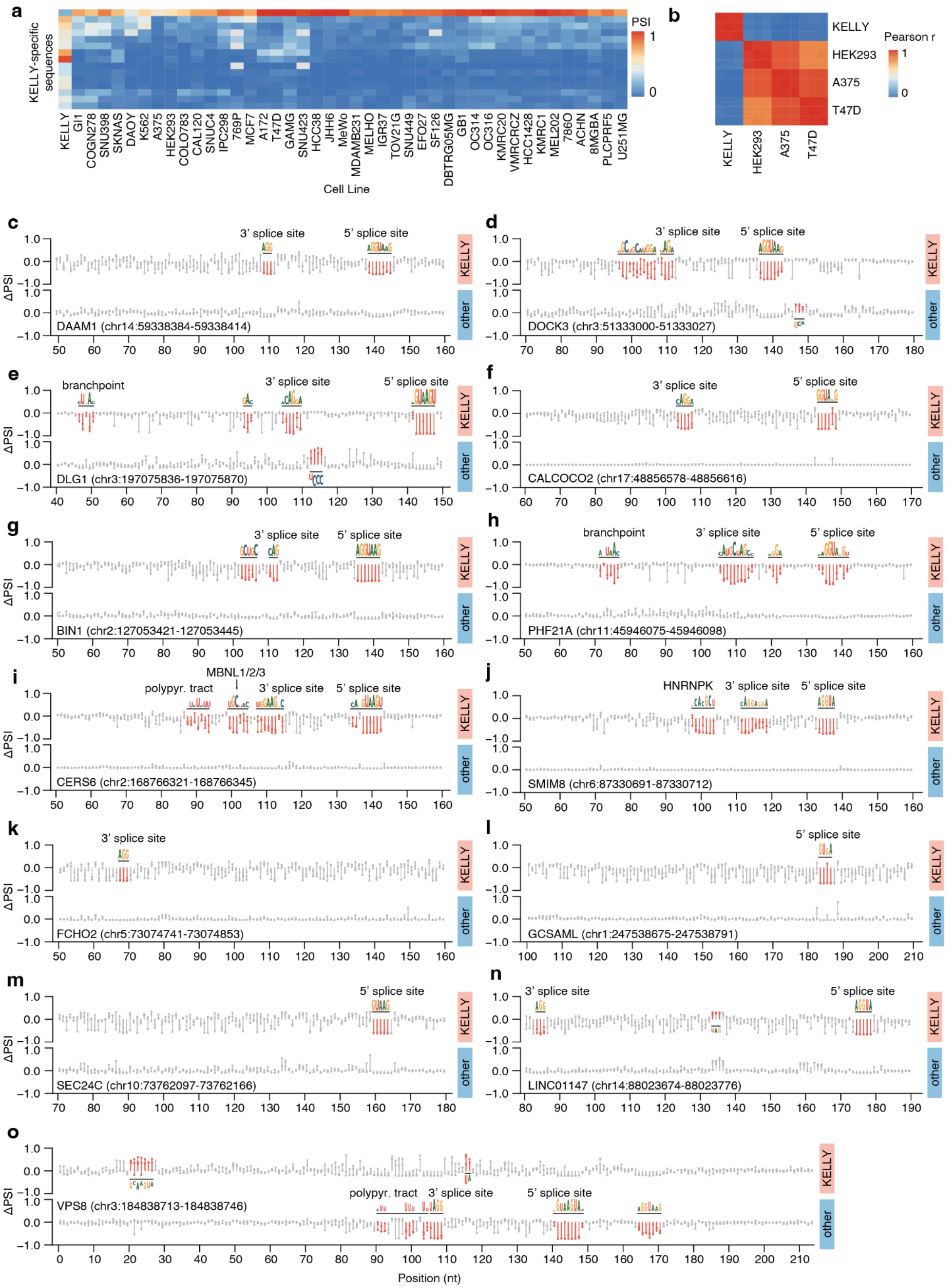

bioRxiv preprint doi: https://doi.org/10.1101/2025.11.05.686847; this version posted November 5, 2025. The copyright holder for this preprint (which was not certified by peer review) is the author/funder, who has granted bioRxiv a license to display the preprint in perpetuity. It is made available under aCC-BY 4.0 International license.

Supplementary Figure 3: Saturation mutagenesis reveals sequence determinants driving cell type-specific splicing

a, Heatmap of fifteen KELLY-specific elements across all cell lines from the unbiased MPRA library, selected to use for saturation mutagenesis. Heatmap color scale indicates PSI, where red represents PSI = 1, and blue represents PSI = 0.

b, Heatmap of Pearson's correlations of PSI between cell lines profiled in the saturation mutagenesis library.

c-o, Effect size ΔPSI for all saturation mutagenesis variants, separated by parent sequence and between KELLY and non-KELLY cells. Sequence motifs are splicing sensitive regions called by our *z*-score filtering approach (Methods).

c, *DAAM1* (chr14:59338384–59338414)
d, *DOCK3* (chr3:51333000–51333027)
e, *DLG1* (chr3:197075836–197075870)
f, *CALCOCO2* (chr17:48856578–48856616)
g, *BIN1* (chr2:127053421–127053445)
h, *PHF21A* (chr11:45946075–45946098)
i, *CERS6* (chr2:168766321–168766345)
j, *SMIM8* (chr6:87330691–87330712)
k, *FCHO2* (chr5:73074741–73074853)
l, *GCSAML* (chr1:247538675–247538791)
m, *SEC24C* (chr10:73762097–73762166)
n, *LINC01147* (chr14:88023674–88023776)
o, *VPS8* (chr3:184838713–184838746)
Polypyr. tract: polypyrimidine tract.

bioRxiv preprint doi: https://doi.org/10.1101/2025.11.05.686847; this version posted November 5, 2025. The copyright holder for this preprint (which was not certified by peer review) is the author/funder, who has granted bioRxiv a license to display the preprint in perpetuity. It is made available under aCC-BY 4.0 International license.

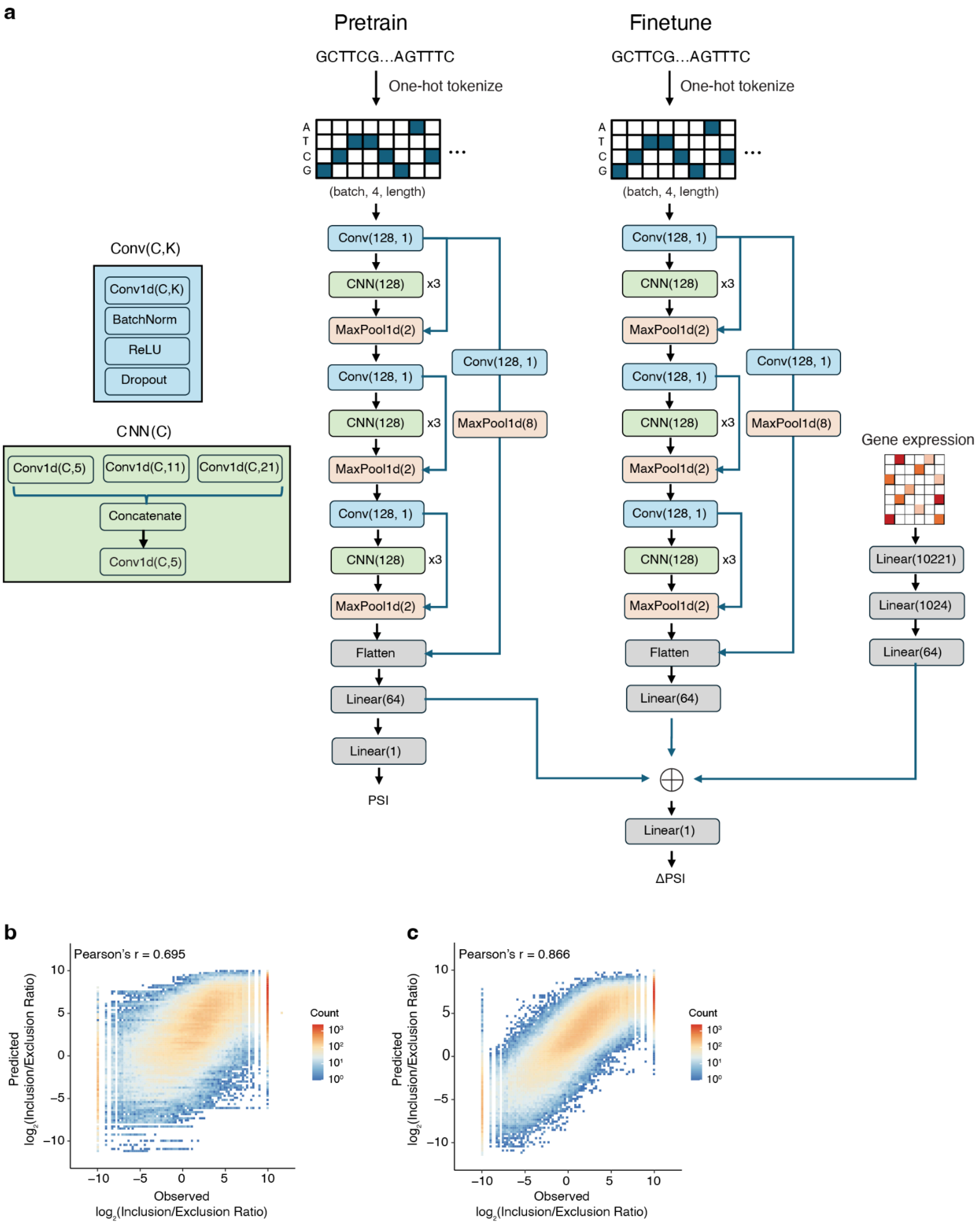

bioRxiv preprint doi: https://doi.org/10.1101/2025.11.05.686847; this version posted November 5, 2025. The copyright holder for this preprint (which was not certified by peer review) is the author/funder, who has granted bioRxiv a license to display the preprint in perpetuity. It is made available under aCC-BY 4.0 International license.

Supplementary Figure 4: Soma model structure and prediction accuracy.
a, Schematic of the Soma model architecture.
b, Scatter of Pearson's correlations between predicted and observed splicing outcomes for a held-out set for unseen sequences using only sequence information.
c, Scatter of Pearson's correlations between predicted and observed splicing outcomes for a held-out set for unseen cell types using only sequence information.

bioRxiv preprint doi: https://doi.org/10.1101/2025.11.05.686847; this version posted November 5, 2025. The copyright holder for this preprint (which was not certified by peer review) is the author/funder, who has granted bioRxiv a license to display the preprint in perpetuity. It is made available under aCC-BY 4.0 International license.

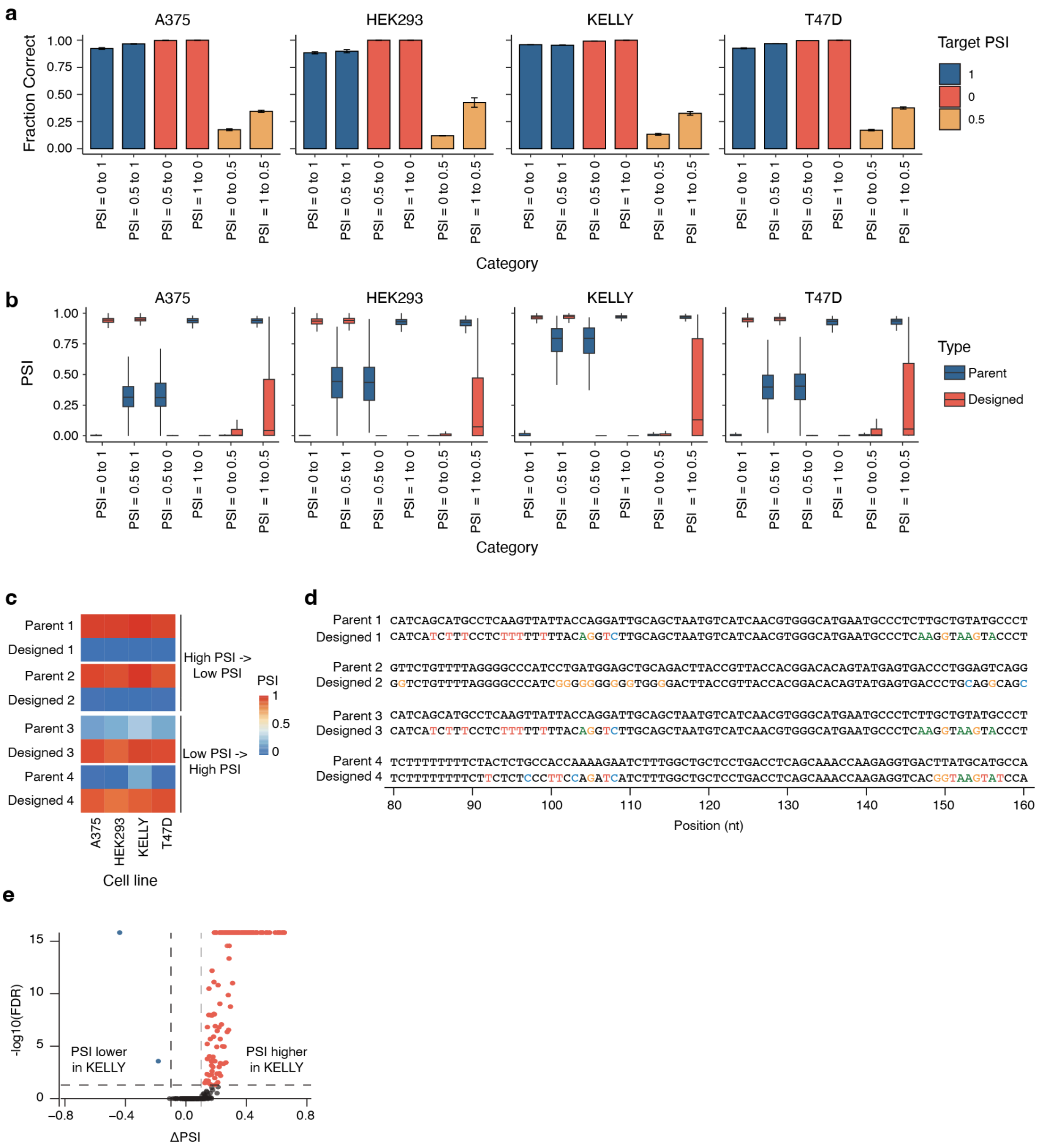

Supplementary Figure 5: Melange accurately generates sequences with user-specified splicing profiles.
a, Fraction of sequences achieving the intended outcome across design categories in the profiled cell lines. Error bars indicate standard deviation across experimental replicates (n = 3).
b, Distribution of PSI values for designed sequences across target categories in the profiled cell lines.

bioRxiv preprint doi: https://doi.org/10.1101/2025.11.05.686847; this version posted November 5, 2025. The copyright holder for this preprint (which was not certified by peer review) is the author/funder, who has granted bioRxiv a license to display the preprint in perpetuity. It is made available under aCC-BY 4.0 International license.

c, PSI values of example sequences from the bin-switching task, for both the parent and designed sequences in the categories {high PSI (>0.8) to low PSI (<0.2)} and {low PSI (<0.2) to high PSI (>0.8)}. Values are averaged across three replicates in each cell line.
d, Example sequences from the bin-switching task. Highlighted nucleotides indicate differences between the parent sequence and the designed sequence.
e, Differential splicing of KELLY-specific designed sequences identified by rMATS, comparing PSI between KELLY and non-KELLY cells. Significant events were defined as $\Delta PSI > 0.1$ and $FDR < 0.05$. Red points indicate higher PSI in KELLY cells, and blue points indicate lower PSI in KELLY cells.

bioRxiv preprint doi: https://doi.org/10.1101/2025.11.05.686847; this version posted November 5, 2025. The copyright holder for this preprint (which was not certified by peer review) is the author/funder, who has granted bioRxiv a license to display the preprint in perpetuity. It is made available under aCC-BY 4.0 International license.

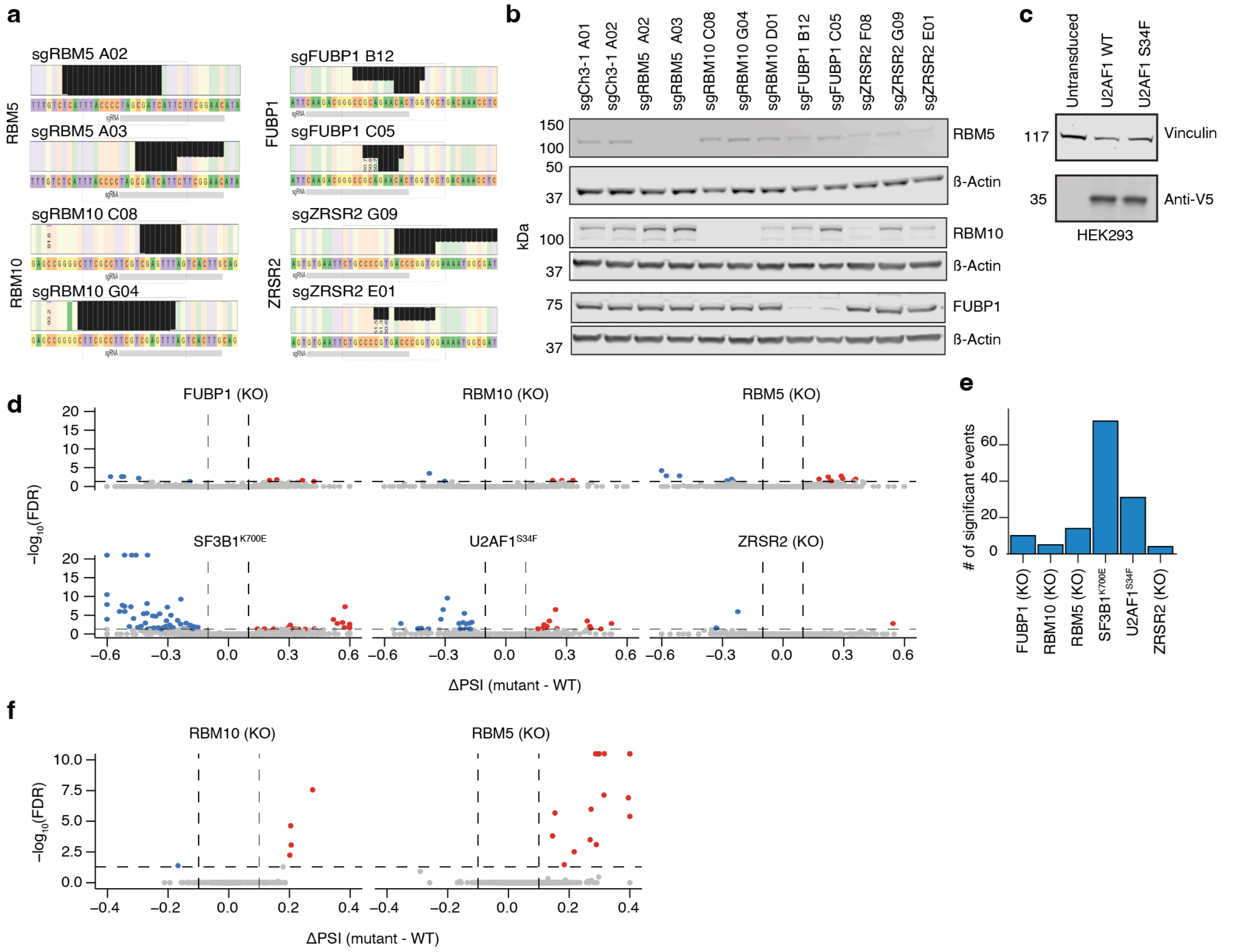

Supplementary Figure 6: Cells harboring cancer mutations display misregulated splicing profiles

a, CRISPR knock-out sites for K562 single cell clones, for *RBM5* KO, *RBM10* KO, *FUBP1* KO, and *ZRSR2* KO cells.

b, Western blotting for RMB5, RBM10, and FUBP1 in each of the single cell K562 clones. Ch3-1 guides are non-targeting sgRNAs. ZRSR2 expression is not shown as no commercial antibodies had sufficient binding affinity.

c, Western blotting for V5-tagged U2AF1^{WT} and U2AF1^{S34F} expression in HEK293. Cells were infected with lentivirus containing a transgene with the corresponding *U2AF1* cDNA sequence.

d, Volcano plots of significant library elements identified per mutant background, for alternative 3′ splice site selection. Significant events have ΔPSI > 0.1 and FDR < 0.05. Significant points in red have higher PSI values in the KO cells than WT cells, and points in blue have lower PSI values in the KO cells than WT cells.

e, Number of significant library elements identified per mutant background, filtered by ΔPSI > 0.1 and FDR < 0.05, for alternative 3′ splice site selection.

f, Volcano plots of significant sequences between mutant and WT paired cell lines in alternative 3′ splice site selection. Significant events have ΔPSI > 0.1 and FDR < 0.05. Significant points in red have higher PSI values in the KO cells than WT cells, and points in blue have lower PSI values in the KO cells than WT cells.

KO: knock-out. WT: wild-type.
